# The sodium pump and digitalis drugs: Dogmas and fallacies

**DOI:** 10.1002/prp2.505

**Published:** 2019-07-19

**Authors:** Amir Askari

**Affiliations:** ^1^ Department of Cancer Biology, College of Medicine and Life Sciences University of Toledo Toledo Ohio

**Keywords:** cardiac glycosides, digitalis, digoxin, Na/K‐ATPase, ouabain, sodium pump

## Abstract

The sodium pump (Na/K‐ATPase) is a plasma membrane enzyme that transports Na^+^ and K^+^ against their physiological gradients in most eukaryotic cells. Besides pumping ions, the enzyme may also interact with neighboring proteins to activate cell signaling pathways that regulate cell growth. Digitalis drugs, useful for the treatment of heart failure and atrial arrhythmias, inhibit the pumping function of Na/K‐ATPase and stimulate its signaling function. In the current field of research on the sodium pump and digitalis drugs, some issues that are commonly accepted to be well established are not so, and this may impede progress. Here, several such issues are identified, their histories are discussed, and their open discussions are urged. The covered unsettled questions consist of (a) the suggested hormonal role of endogenous digitalis compounds; (b) the specificity of Na/K‐ATPase as the receptor for digitalis compounds; (c) the relevance of the positive inotropic action of digitalis to its use for the treatment of heart failure; (d) the conflicting findings on digitalis–induced signaling function of Na/K‐ATPase; and (e) the uncertainties about the structure of Na/K‐ATPase in the native cell membrane.

## 1 INTRODUCTION

No field of research is free of controversies, and disagreements usually stimulate progress. In research fields that have a long history, however, some disagreements among one generation of scientists are simply forgotten by the next generations, thereby leading to common assumptions of "established" or "known" facts that are not so. These dogmas and fallacies impede progress. Digitalis drugs (cardiac glycosides) were introduced into Western medicine in the latter part of the 18th century,^1^ and since then have been used primarily for the treatment of congestive heart failure and atrial arrhythmias. It was not until the second half of the 20th century that these drugs were found to be inhibitors of the membrane–bound Na/K‐ ATPase or the sodium pump.^2^, ^3^ Since then these drugs have also become important tools in extensive and continuing studies on the structure–function relationship of the sodium pump. This long history, spanning several generations of investigators, has permitted the development of a good number of dogmas and fallacies that are now masquerading in the literature as “established” or “known” facts. The purpose of this review is to identify some of these and outline their histories. Needless to say, the aim is not to end the existing uncertainties, but simply to urge the active researchers of the field not to overlook the unknowns, and not to mislead their students and the newcomers to the field. The falsely labeled “established” issues that I have identified are listed below not in order of importance; all are equally egregious.

### 1.1 Endogenous digitalis compounds are hormones

In late 1950s, just about the time that Skou^2^, ^3^ discovered that Na/K‐ATPase was indeed the digitalis–sensitive sodium pump that had been sought after for decades,^4^ it was also suggested by several laboratories that there may be a circulating factor in the plasma of experimental animals and man capable of inhibiting the sodium pump.^5^, ^6^, ^7^ Thus the possibility of the existence of endogenous digitalis–like compounds was born. This was debated for decades as an interesting hypothesis with no conclusion about either the existence or the nature of the factor until 1989‐1991, when the results of a collaborative research between scientists at a pharmaceutical company (Upjohn) and those at the University of Maryland were published claiming the isolation, purification, and identification of a ouabain–like compound from human plasma.^8^, ^9^, ^10^, ^11^, ^12^, ^13^, ^14^ These papers which gave a chemical face to the endogenous factor, and the continued work of this group^15^ prompted a predictable response from the investigators of the field; that is, there were pros and cons,^16^, ^17^, ^18^ and severe criticisms.^19^ What was unexpected, and perhaps irresponsible, was the response of the scientific establishment to the findings of the the Upjohn/Maryland group. Very soon after the initial publications of this group and in spite of the criticisms of the well–known working investigators of the field, Lancet^20^ wrote an editorial entitled “Welcome to the ouabain‐a new steroid hormone”. Within a decade, The American Heart Association’s “Hypertension Primer” was referring to ouabain as a hormone whose levels regulated blood pressure and possibly the pathogenesis of the heart failure, even though no new experimental evidence had been published to refute previous criticisms. What is understandable is the tenacity of the Upjohn/Maryland team in advocating the hormonal role of ouabain. The Upjohn Company had no commercial interest in pushing a long–known drug as a hormone, and soon washed its hand off the project. But the involved scientists both at Upjohn and Maryland, who had put “blood, sweat, and tears” into their extensive research are entitled to not being entirely objective in this controversy, and are still campaigning to sell ouabain as a hormone.^22^ What is puzzling, however, is that so many other investigators doing any kind of research on the sodium pump or digitalis have bought into the proposal that there must be “digitalis‐like hormones” in spite of several publications that point out the necessity of being skeptical, at the very least, about the existence of such hormones.^23^, ^24^, ^25^ To make matters more complex, there are also serious disagreements among the believers on the nature of any such hormones.^22^, ^23^, ^26^, ^27^ Why do those of us who have not spent a day trying to find experimental support for or against endogenous digitalis keep citing the existence of such hormones? I think that we are just captivated by the prospect that we are not only working with an enzyme (the sodium pump) that is physiologically important, but that the enzyme’s inhibitors may also be physiologically important. Under the circumstances, I suggest that the casual assumption of the existence of digitalis–like hormones is indeed an unjustified and misleading fallacy.

### 1.2 Sodium pump is the only known receptor for digitalis drugs

This is perhaps the most entrenched dogma of this field of research. When it was shown by Schatzmann^28^ that cardiac glycosides inhibit the active transports of Na^+^ and K^+^, and this was confirmed by others^4^; and when a few years later Skou^3^ showed that his newly discovered Na/K‐ATPase was indeed inhibited by the same cardiotonic steroids, there was no reason to assume that the sodium pump would be the only receptor for these old drugs. On the contrary, examination of history of the extensive studies on the metabolic effects of cardiac glycosides prior to the discovery of Na/K‐ATPase would clearly show that multiple cellular receptors for these drugs had been suggested.^29^, ^30^ Even in 1971, well after the discovery of the Na/K‐ATPase, a thorough review of the literature on the subcellular basis of cardiac glycoside action by leading investigators concluded that though the singularly established effect of these drugs on a well–defined entity is their inhibitory action on Na/K‐ATPase, other findings indicate that this effect is probably not the only action of the drugs.^31^ In two subsequent reviews published about a decade later by well–respected investigators,^32^, ^33^ the possibility of digitalis receptors other than Na/K‐ATPase was also clearly pointed out. Thereafter, however, this possibility gradually disappeared from the literature. Nowadays, nearly everyone in the field perpetuates the dogma of the sodium pump being the selective receptor for digitalis drugs. The problem is that recent studies also indicate that this dogma should be discarded. Consider the interesting findings suggesting the pleotropic actions of digitalis drugs,^34^, ^35^ and the elegant studies of a respected laboratory^36^ that clearly indicate direct bindings of bufalin, ouabain, digoxin and digitoxin to the transcriptional regulator steroid receptor coactivators 3 and 1 (SRC‐3 and SRC‐1) causing inhibition of cell proliferation. In view of both old and new evidence for nonspecificity of cardiac glycosides, why do the great majority of active laboratories of the field keep repeating the dogma on their specificity? I suggest that the honest answer is that in the competitive market of securing grant support for keeping one’s research alive (at least in USA), it is simply more convenient to ignore complications that are not widely known. This may not be a bad strategy for the survival of this or that laboratory, but it can hardly be justified as a legitimate way of advancing the field.

### 1.3 The positive inotropic action of digitalis is the basis of its use for the treatment of congestive heart failure

Such a statement is often found in the introduction or discussion of any paper dealing with Na/K‐ATPase and digitalis, especially when the paper does not deal with the treatment of heart failure! The statement is usually intended to convey potential clinical importance of any type of research on sodium pump/digitalis interaction. The truth is, however, that in the long history of the use of digitalis for treatment of heart failure there has never been unanimity of opinion on the importance of the drug's positive inotropic action in its cardiac effects. In fact the opinions of clinical scientists on this issue have repeatedly changed with time as new evidence has become available. To see where we stand now a brief review and time‐line of the events is instructive.

In the often–cited monograph of Withering^1^ which introduced foxglove into Western medicine for the treatment of dropsy (swelling of the limbs), he considered the drug as a diuretic. Between this introduction and the first few decades of the 20th century, distinguished physician scientists argued that the drug's efficacy was due to its direct action on the heart,^37^ but many also argued otherwise.^38^ This was the state of affairs until it was shown^39^, ^40^ that ouabain and digoxin increased the force of contraction of the isolated cat papillary muscle, and concluded that the positive inotropic actions of these drugs were the basis of their efficacy. Though advocating this conclusion, the same investigators were also quite aware of the long‐ standing observations that challenged this view, and considered “vagal factors” contributing to digitalis efficacy in the treatment of heart failure^41^, ^42^While the view on the primacy of the drug's positive inotropic effect predominated during the second half of the 20th century,^43^, ^44^ there certainly was no unanimity among pharmacologists. Solid arguments, based on studies in man were presented to indicate that the vagal effects of digitalis may be just as important, or more so, than its positive inotropic action.^45^, ^46^ This controversy persisted until the publication of the Digitalis Investigation Group DIG) trial which had little to do with the mechanism of digitalis action, but was designed to resolve centuries of uncertainties on the long–term efficacy and safety of digoxin by a modern well–designed clinical trial.^47^ The results of this study, however, resolved very little about the aim of this study. Both advocates and opponents of digitalis use found some support in the initial analyses of the data^48^, ^49^ which showed no beneficial effects of digoxin on mortality and modest effects on morbidity. More importantly, several subsequent retrospective analyses of the DIG data altered the initial conclusions and led to strong recommendations for expanded use of digoxin for treatment of heart failure at the lower range of doses that were used in the DIG trial.^50^, ^51^, ^52^ Regardless of the impact of this trial on the present and future use of digoxin, the trial's findings reveal how far we are in elucidating the mechanism of digoxin efficacy in treatment of heart failure in man. Consider that the post hoc data analysis indicates that digoxin reduces mortality if the dosage is such that the blood levels do not exceed 0.64‐1.15 nmol/L digoxin.^50^ Though the evidence is strong enough to indicate that at these levels digoxin is a neuro‐hormonal modulator,^46^ we may ask if there is any evidence to support the possibility of positive inotropic effects of such low blood levels in man? To assess this possibility we need to have two sets of data about the human myocardial Na/K‐ATPase: First, the composition of enzyme isoforms in human ventricular muscle; and second, the sensitivities of the existing isoforms to digoxin. Significant work on these issues was done by several capable laboratories^53^, ^54^, ^55^, ^56^; and was reviewed soon after.^57^, ^58^ Careful examination of this literature would indicate that in spite of valiant efforts of several laboratories, no consistent answers to the questions of isoform compositions of human heart and their digitalis sensitivities exist. Most of this inconsistency is likely due to the problem that digitalis sensitivities of human cardiac preparations were determined by drug bindings to the enzyme under nonphysiological conditions; an issue that has been discussed elsewhere.^59^ I suggest that to settle whether or not 0.64‐1.5 nmol/L digoxin produces any measurable positive inotropy in the human heart, the active workers of the field should roll up their sleeves and measure the relative values of human cardiac isoforms and their digoxin sensitivities under Na/K‐ATPase turnover conditions. Until such studies are done we should stop repeating the questionable statement in the heading of this section, and assume as dictated by Occum's razor, that at serum levels of 0.64‐1.15 nmol/L, digoxin's beneficial effects on heart failure patients are primarily due to its vagal actions.^50^

### 1.4 The cell signaling functions of Na/K‐ATPase provide support for (or against) previously inexplicable digitalis effects

This is the newest of the field's fallacies covered here, with the shortest history. It is primarily due to our inability to appropriately evaluate the expansion of the field in novel directions. During a few decades after the discovery of Na/K‐ATPase,^2^ some isolated studies suggested that the sodium pump regulates cell growth. It was not until the 1990s, however, that attention was focused on the mechanism of such regulation. My laboratory^60^and that of Aperia^61^ were the first to present evidence indicating that digitalis interaction with the Na/K‐ATPases of cardiac myocytes or epithelial cells activate growth stimulatory pathways independent of the drug's inhibition of the ion pumping function of the enzyme and changes in intracellular ion concentrations. These early studies which have been appropriately reviewed by the two laboratories^62^, ^63^ led to extensive subsequent studies of the signaling functions of Na/K‐ATPase by several laboratories. While these studies have clearly advanced the field in novel directions, they have also led to a common problem of many new and developing research areas; that is, the fact that novel findings need to be verified by independent repetitions. Regrettably, the literature of the cell signaling function of the sodium pump during the past two decades has been rich in apparent irreproducibility of results. Perhaps the most well‐known of these is the strongly advocated hypothesis of direct interaction of Na/K‐ATPase with Src,^64^, ^65^ which has been challenged by the apparent inability of others to reproduce the supporting experiments of the hypothesis.^66^, ^67^, ^68^, ^69^ In spite of this unsettled controversy created by the works of several competent laboratories, the Src/sodium pump interaction is often cited in the literature as established fact, not only by the original proposers of the hypothesis, but also by others to support other dubious proposals.^22^, ^23^ As it has been aptly noted^62^ about the signaling function of Na/K‐ATPase, “contributions to this field have so far come from a limited number of research groups”. I suggest that until multiple laboratories attempt the repetitions of critical experiments, and the test of time permits arriving at a consensus on what observations are reproducible, we should be cautious in accepting the proposed conclusions of studies on the signaling function of Na/K‐ATPase, as appealing as such proposals may be.

### 1.5 The reaction mechanism of Na/K‐ATPase is adequately explained by the Albers‐Post scheme (or the E1‐E2 cycle) of the enzyme monomer(the α,β,γ protomer)

**Figure 1:**
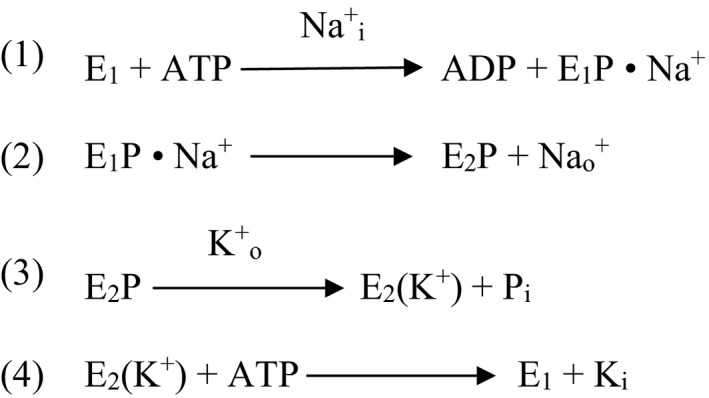
The Albers‐Post cycle of Na/K‐ATPase activity. Na_i_^+^ and K_i_^+^ are intracellular ions, and Na_o_^+^ and K_o_^+^ are extracellular ions

I end my listing of the undeserved “established” facts of the field with this often–repeated statement about the reaction mechanism of the sodium pump. After the discovery of Na/K‐ATPase,^2^ it was indeed well‐established through the works of numerous laboratories^70^ that two subunits of the enzyme (*α* and *β*) are essential for function, a third subunit (γ) regulates function, and that the enzyme performs the coupled transports of Na^+^ and K^+^ across the membrane by the Albers‐Post cycle as depicted in Figure 1. However, it was certainly not established that the enzyme monomer (the protomer) could go through this cycle. There used to be a time, say within the first fifty years after the discovery of the enzyme, that there were intense debates among the leading laboratories as to whether the monomer (protomer) or an oligomer (diprotomer or tetraprotomer) went through the Albers‐Post cycle.^70^, ^71^, ^72^ The disagreements were primarily due to the fact that many findings suggested that the two ATP sites of the Albers‐Post cycle (reactions 1 and 4 of Figure 1) were indicative of multiple ATP sites in the functional unit of the pump, rather than different affinities of the same site.^71^ Amazingly, these disagreements and debates started to disappear from the literature soon after the turn of the 20th century; not because the disagreements were resolved, but because most of the investigators working on this issue either retired or simply stopped debating. Why the latter? Perhaps this was due to the rise of the importance of “translational biomedical research” at about the same time, and the fact that it became more difficult to obtain grant support for attempting to resolve the reaction mechanism of an enzyme than to find a cure for a disease. So, the end result is that the current active investigators of the field seem to be ignoring the existence of an unresolved issue. It is fortunate, however, that the negligence is not total. Some have tried to keep the debate alive.^73^, ^74^ It is also gratifying that in the recently published memoirs of the late discoverer of the enzyme, he confirmed his belief that the dimer–monomer issue remained unresolved in spite of some crystallographers’ contrary opinions. I suggest that when the reaction mechanism of the pump needs to be presented in a publication, even if the mechanism is not the primary focus, a brief reference to nearly half century of unresolved disagreements on the monomer–oligomer issue is mandatory.

## 2 CONCLUDING REMARKS

Old fields of research such as the one covered here have many dogmas and fallacies. Here, I have listed and discussed the histories of six that I believe are of potential harm to the progress of this field. In Table 1, for the convenience of the readers, I have summarized the most important contradictory articles for each of the six areas of controversies. It may seem that by focusing on these shortcomings, I am being too harshly critical of the collective achievements of the scientists of this research area. This is not so; I am advocating the candid admission of our inevitable shortcomings. To quote from one of my favorite prominent scientists when he was reflecting on the nature of the work of a scientist,^76^ “contrary to the general belief, there are no answers in science. Any answer is only ever a suggestion, another opportunity to wonder, that will eventually be revised.”

**Table 1:** Important articles indicating unresolved disputes on the discussed controversies

Controversial issue	Reference numbers of the cited articles
1) Hormonal roles of endogenous digitalis compounds.	16, 19, 22, 23, 24, 25, 26, 27
2) Is Na/K‐ATPase the only receptor for digitalis?	30, 31, 32, 33, 36
3) Is positive inotropic action of digitalis the basis of its efficacy in treatment of heart failure?	39, 40, 42, 46, 50
4) Conflicting findings on cell signaling functions of Na/K‐ATPase.	60, 61, 62, 64, 67, 68, 69
5) Conflicting views on the oligomeric structure of Na/K‐ATPase in the native membrane.	70, 71, 72, 73, 74
